# LGR4, a G Protein-Coupled Receptor With a Systemic Role: From Development to Metabolic Regulation

**DOI:** 10.3389/fendo.2022.867001

**Published:** 2022-05-30

**Authors:** Joanna Filipowska, Nagesha G. Kondegowda, Nancy Leon-Rivera, Sangeeta Dhawan, Rupangi C. Vasavada

**Affiliations:** Department of Translational Research and Cellular Therapeutics, Arthur Riggs Diabetes and Metabolism Research Institute City of Hope, Duarte, CA, United States

**Keywords:** LGR4, GPCRs (G protein-coupled receptors), Wnt signaling, RANKL (Receptor Activator for Nuclear Factor k B Ligand), inflammation, NFκB - Nuclear Factor κB, LGR4-ECD, miRNA34 a/c

## Abstract

Leucine-rich repeat-containing G protein-coupled receptor 4 (LGR4/GPR48), a member of the GPCR (G protein-coupled receptors) superfamily, subfamily B, is a common intestinal crypt stem cell marker. It binds R-spondins/Norrin as classical ligands and plays a crucial role in Wnt signaling potentiation. Interaction between LGR4 and R-spondins initiates many Wnt-driven developmental processes, e.g., kidney, eye, or reproductive tract formation, as well as intestinal crypt (Paneth) stem cell pool maintenance. Besides the well-described role of LGR4 in development, several novel functions of this receptor have recently been discovered. In this context, LGR4 was indicated to participate in TGFβ and NFκB signaling regulation in hematopoietic precursors and intestinal cells, respectively, and found to be a new, alternative receptor for RANKL (Receptor Activator of NF kappa B Ligand) in bone cells. LGR4 inhibits the process of osteoclast differentiation, by antagonizing the interaction between RANK (Receptor Activator of NF kappa B) and its ligand-RANKL. It is also known to trigger anti-inflammatory responses in different tissues (liver, intestine, cardiac cells, and skin), serve as a sensor of the circadian clock in the liver, regulate adipogenesis and energy expenditure in adipose tissue and skeletal muscles, respectively. The extracellular domain of LGR4 (LGR4-ECD) has emerged as a potential new therapeutic for osteoporosis and cancer. LGR4 integrates different signaling pathways and regulates various cellular processes vital for maintaining whole-body homeostasis. Yet, the role of LGR4 in many cell types (e.g. pancreatic beta cells) and diseases (e.g., diabetes) remains to be elucidated. Considering the broad spectrum of LGR4 actions, this review aims to discuss both canonical and novel roles of LGR4, with emphasis on emerging research directions focused on this receptor.

## LGRs, Members of GPCR Subfamily B: Molecular Characteristics and Expression Pattern

LGR4 (Leucine-rich repeat (LRR)-containing G-protein-coupled receptor 4) or GPR48 (G-protein- coupled receptor 48), together with LGR5 and 6, is a member of the receptor GPCR superfamily, LGR subfamily B (II), highly homologous to glycoprotein hormone receptors, e.g., LHR (Luteinizing Hormone Receptor), TSHR (Thyroid Stimulating Hormone Receptor) (1–3). LGR4, 5, and 6, are critical regulators of embryonic development, and also have been shown to contribute to several cancers (4–6). They are responsible for adult stem cell maintenance *in vivo* and stem cell survival *ex vivo* (7, 8), *via* direct interaction with ZNRF3 (Zinc And Ring Finger 3), LRP5/6 (LDL Receptor Related Protein 5) and Frizzled receptors, the components of the Wnt signaling pathway (9).

**Figure 1:**
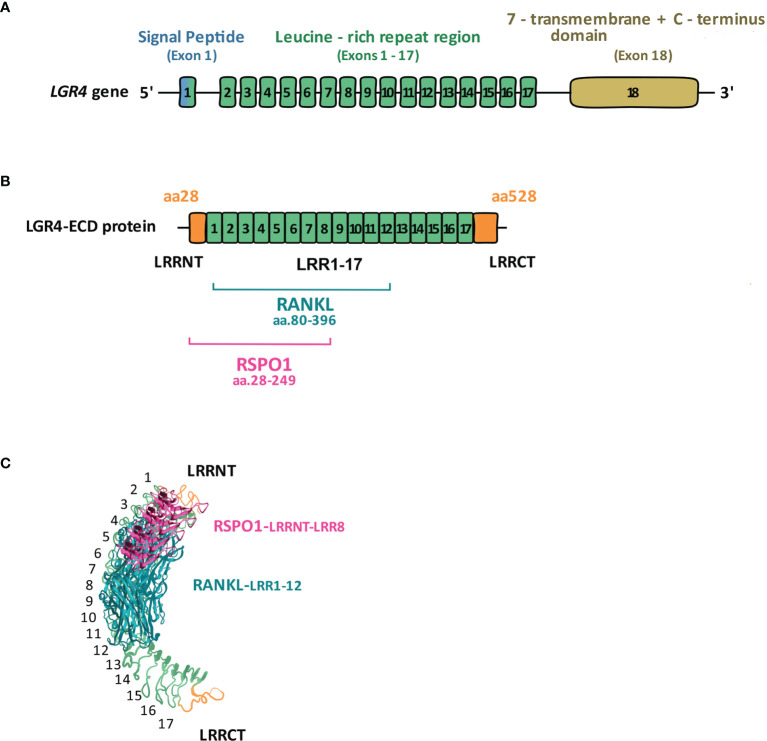
LGR4 gene and protein structure. **(A)** LGR4 gene contains three main domains: leucine-rich repeats-containing N-terminal domain encoded by exons 1-17, responsible for binding ligands (RSPOs, NORRIN, RANKL); seven-transmembrane (7TM) domain encoded by exon 18, anchoring the receptor within the cell membrane; C-terminal domain encoded by exon 18, responsible for signal transduction. **(B)** Full-length LGR4 is composed of 951 aa, of which LGR4-ECD comprises aa. 28-528. Within LGR4-ECD aa.28-249 (LRRNT-LRR8) are responsible for RSPO1, whereas aa.80-396 (LRR1-12) for RANKL binding [modified from reference (11)]. **(C)** 3D structure of LGR4-ECD binding to RSPO1 and RANKL [modified from (12)].

Structurally, all three LGRs (4–6) have highly conserved seven-transmembrane (7TM), rhodopsin-like regions and either 18 (LGR4 and 5) or 13 (LGR6) horseshoe-like leucine-rich repeats in the N-terminal (NT) extracellular domain responsible for ligand-binding, flanked with cysteine-rich sequences known as LRRNT and LRRCT (10). The intracellular region of LGRs ends with the C-terminal (CT) sequence, and is responsible for ligand-mediated signal transduction **(** **Figure 1** **).**

All three members of the human GPCR subfamily B share some genetic similarities. *LGR4* and *5* have 46% homology, whereas *LGR4* and *6* share 44% sequence similarity. *LGR5* and *6* are molecularly closer to one another and share 54% identity (13). All three receptors are also evolutionarily conserved across vertebrate species, e.g., human *LGR4*/*LGR5*/*LGR6* share 90%, 82%, and 84% similarity with their mouse orthologs: *Lgr4*/*Lgr5*/*Lgr6*, respectively (13). Two paralogs (*lgr4* and *lgr6*) are also found in Teleosts (13), the Actinopterygii intraclass representing 96% of all the existing fish species. Besides vertebrates, the orthologs of Lgrs are also found in invertebrates, e.g., *Dlgr2 i*n a fruit fly, *Drosophila melanogaster*, is involved in exoskeleton morphogenesis and hardening (14). As indicated by the NCBI gene database, in humans, LGR4 is located on chromosome 11, in the domestic mouse (*Mus musculus*) on chromosome 2 and in fruit flies on the sex chromosome X.

UCSC Genome browser data (published in 2019) indicates *LGR4* is commonly expressed in different human tissues, e.g. kidney, heart, gastroesophagus, pancreas, achieving the highest expression level in the ovary. A comprehensive atlas of LGR4 protein expression in different mouse and human tissues was generated by Yi et al. (15). Using custom-made antibodies against LGR4 and employing immunostaining technique, these authors showed that LGR4 is present in the epidermis and hair follicle of the skin, kidney, pancreatic β-cells, and epithelial cells of both male and female reproductive organs. Besides assessing tissues from healthy subjects, Yi et al. also examined colon tumor samples extracted from colon cancer-suffering patients, confirming high expression of LGR4 in the biopsies of this type of cancer. The role of LGR4 expression in development and progression of different types of cancer has also been recently reviewed by Ordaz-Ramos et al. (4).

## Lgr4 and Wnt Signaling

Traditionally, members of the LGR subfamily are described as Wnt (Wingless-related integration site) signaling facilitators, since they enable signal transduction by the WNT ligands. In this context, the most critical is interaction of the N-terminus of LGRs (the extracellular domain) with R-spondins (Roof plate specific spondins, RSPOs), first defined as LGR4 and 5 ligands in 2011 (16, 17). The RSPO family comprises of four members: RSPO1 to 4, sharing between 40-60% homology with one another (18, 19). Structurally, RSPOs interact with LGRs *via* their Furin-like domains. All four RSPOs can induce canonical Wnt signaling *via* all three members of the GPCR subfamily B (19). RSPO3 is capable of inducing the non-canonical planar cell polarity Wnt pathway during gastrulation, head cartilage formation, and craniofacial development (20). Notably, RSPOs typically do not possess signaling activity on their own and require WNT ligands to execute their functions (21).

Traditionally, the WNT-mediated signal potentiation by LGRs is possible due to RSPOs interaction with E3 ubiquitin homologous ligases RNF43 (Ring finger 43) or ZNRF3, which inhibit activation of Frizzled- the WNT protein receptor (22). Interaction of RSPOs with the extracellular domains of RNF43 and ZNRF3 leads to their clearance from the cell membrane and thus enables Wnt signaling to proceed (22, 23). Interestingly a recent paper by Park et al. (24), indicates LGR4 and LGR5 have distinct roles in Wnt signaling potentiation. LGR4 does not require RSPOs to interact with ZNRF3 and RNF43 ligases and LGR5 does not interact with either of the ligases and is thus a weaker activator of this pathway than LGR4. Besides RSPOs, Wnt signaling potentiation *via* LGR4 can also be mediated by NORRIN (Norrie Disease Protein), a ligand structurally distinct from RSPOs which does not interact with either LGR5 or 6 (25).

## LGR4 Function in Health and Disease

### Development

Wnt signaling potentiation is essential for proper development as it regulates cell migration, differentiation and polarization during embryogenesis (26). LGR4, together with LGR5 and 6 are involved in the maintenance of stem cells in different tissues, e.g. skin (hair follicle) (27) or intestine (7). LGR4 regulates the formation of the kidney, gut, and skin epithelium (28). Lgr4 (as well as Lgr5, but not Lgr6) null mice exhibit intrauterine growth retardation and neonatal lethality (29), indicating its critical role in development. Many phenotypes resulting from *LGR4* deficiency were also observed in humans. Based on genome-wide association (GWAS) studies, Styrkarsdottir and colleagues (30) found a non-sense mutation within *LGR4* (c.376C.T) which results in multiple impairments, e.g. reduced birth weights, electrolyte imbalance, late onset of menarche and decreased levels of testosterone (15, 30). Similar effects of this mutation were observed in mice.

### Tissue-Specific Role

In the context of canonical Wnt signaling, Luo et al. (31) demonstrated that LGR4 is involved in the formation of the skeletal system. Using a whole-body mouse *Lgr4* knockout, these authors showed that the process of osteo-, but not chondro-genesis is severely impaired in mice lacking *Lgr4*, both during development and in postnatal life. Mechanistically, Luo et al. demonstrated that LGR4-mediated Wnt signaling targets ATF4 expression *via* cAMP-PKA-CREB, to drive osteogenesis. Most recently, Mancini et al. (32) described the importance of LGR4 in puberty. By using GWAS analyses, these authors identified 3 rare missense variants in *LGR4* (NM_018490.3: c.286A>G (rs757351670) p.Ile96Val; NM_018490.3: c.1087G>T (rs117543292) p.Gly363Cys; and NM_018490.3: c.2531A>G (rs34804482) p.Asp844Gly) in 6 unrelated families, in which 17 individuals were diagnosed with delayed puberty. Further, animal (mouse- and zebrafish-based) studies by these authors revealed *lgr4* to play a significant role in formation and migration of the Gonadotropin-releasing hormone expressing neurons (GNRH), which control the secretion of reproductive hormones from the pituitary gland. In the study of Mancini et al., *Lgr4* knockout mice exhibited a significant delay in the onset of puberty, similar to human subjects carrying *LGR4*-related mutations. This suggests a conserved role of LGR4 in puberty regulation across species.

### In Disease

Both, *LGR4* deficiency as well as overexpression, may lead to diseases. Yi et al. (33) suggested *LGR4* deficiency, resulting from the deletion of chromosome 11 regions 11p12–11p14, to be crucial for development of a human genetic syndrome known as WAGR (Wilm’s tumor, aniridia, genitourinary anomalies, and intellectual disability), associated with multiple organ (kidney, eyes) abnormalities and intellectual disability.

As mentioned before, when overactivated later in life, LGR4 can contribute to cancer (4, 15). Yue et al. (6) showed that LGR4 participates in breast cancer progression by stimulating increased Wnt signaling, such that high levels of LGR4 in breast tumors correlate with a patient’s poor prognosis. Corresponding to this, knockdown of *LGR4* specifically in breast cells led to reduced tumor growth and invasiveness *in vitro* and *in vivo*, together with a decrease in the number of functional cancer stem cells. Recently, Zeng et al. (34) defined LGR4 as a relevant prognostic marker in serous ovarian cancer, correlating its high expression levels with poor prognosis in Chinese population. LGR4 overexpression also leads to squamous cell carcinoma (35).

Some cancers, e.g. multiple myeloma employ atypical overexpression of LGR4 on plasma cells (a type of B cell responsible for antibody production, in the bone marrow). B cells normally do not express LGR4. However, when overexpressing LGR4, plasma cells take over RSPOs produced by pre-osteoblasts, to over-activate Wnt signaling and this leads to progression of multiple myeloma (36).

## Tissue-Specific LGR4 Signal Transduction Mechanisms

Besides direct effects of LGR4 on the Wnt pathway, many reports indicate the involvement of this receptor in the crosstalk between Wnt and other pathways, e.g., NFκB (Nuclear Factor-kappa-light-chain-enhancer of activated B cells). Using intestinal cells, Lai et al. (10) recently observed that the intracellular domain of LGR4 and 5 can also trigger N-terminus-(ligand-binding)-independent NFκB signaling on their own. Li et al. (37), showed that LGR4 plays a protective role against liver injury. These authors demonstrated that functional LGR4 is expressed in mature hepatocytes and that RSPO1 protects hepatocytes from Tumor Necrosis Factor-α-induced cell death. When *Lgr4* is specifically deleted in hepatocytes, the liver becomes more susceptible to acute injury (see section 7). The authors concluded that LGR4 protects hepatocytes from injury by inhibiting NFκB signaling. As shown by Han et al. (38), Wnt signaling potentiated by RSPO2 and LGR4 also interacts with Transforming Growth Factor beta (TGFβ) signaling *via* Follistatin (FS) (TGFβ antagonist) to drive early myogenesis. These authors showed that LGR4 is critical for a proper myogenic differentiation mediated by RSPO2. Using a mouse model, *in vitro* and *in vivo*, Han et al. showed that there exists a positive feedback loop between RSPO2, LGR4 and FST in the context of myogenesis, where FST enables Wnt signaling *via* RSPO2 and LGR4 at the same time being a primary target gene of their interaction. Recently, Wang et al. (39) revealed an alternative function of LGR4-RSPO binding related to early mammalian hematopoiesis regulation, both *in vitro* and *in vivo*, which is unrelated to Wnt. These authors have shown that LGR4 targets TGFβ signaling to modulate human pluripotent stem cell (hPSC) hematopoietic differentiation. Deletion of *Lgr4*, but not *Lgr5*, is detrimental for mesoderm development and therefore disables hematopoietic differentiation both *in vitro* and *in vivo*. Wang et al. demonstrated that RSPO1-3, but not RSPO4 interact with LGR4 to promote hematopoietic differentiation. Interestingly, they also showed some differences in receptor binding capability, with RSPO2 acting solely *via* ZNRF3 independently of LGR4, as opposed to RSPO1 and 3. Capability to manipulate LGR4 and RSPOs in the context of hematopoiesis may facilitate the large-scale generation of functional Hematopoietic Stem Cells (HSCs) for potential clinical applications. The study by Luo et al. (11) performed on osteoclasts showed that at the intracellular level, LGR4 can activate Gαq pathway to inhibit GSK3β (Glycogen Synthase Kinase 3 Beta) phosphorylation independently of Wnt signaling, thus preventing NFATC (Nuclear Factor of Activated T-Cells)-mediated osteoclastogenesis (see section 5). In invertebrates (e.g., *D. melanogaster*), DLGR2, the equivalent of LGR4, interacts with bursicon proteins: BURS/PBURS, activating cAMP pathway (14, 40). These reports altogether suggest that LGR4 participates in multiple signaling pathways, dependent on tissue and cell type.

## LGR4, a Novel Receptor for RANKL (Ligand of Receptor-Activator of NFκB)- New Pathway With Clinical Implications

**Figure 2:**
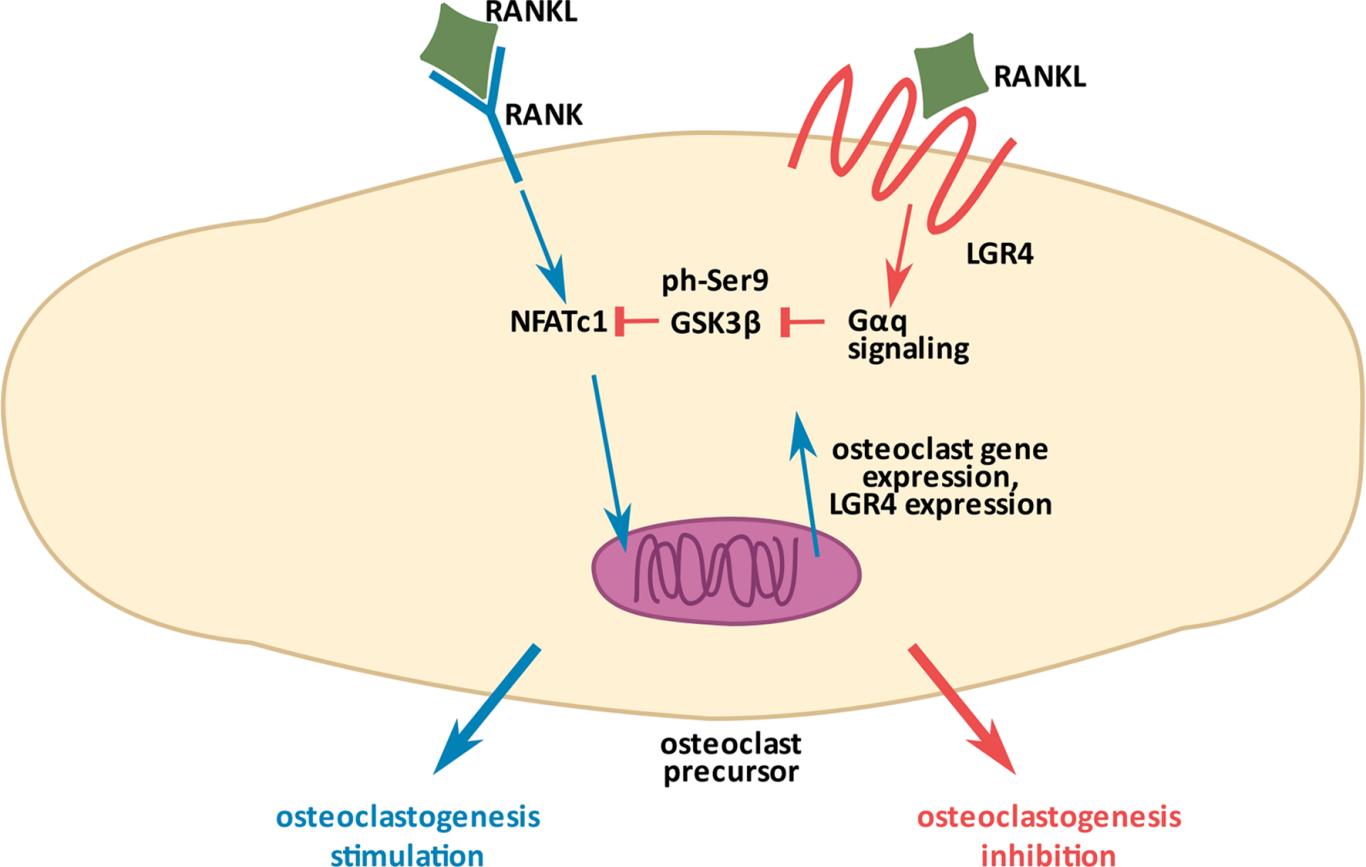
Molecular mechanism of osteoclastogenesis inhibition by LGR4. RANKL (Ligand of Receptor-Activator of NFκB), a common ligand for the two receptors, RANK (Receptor-Activator of NFκB) and LGR4, has opposite effects on osteoclastogenesis. RANKL/RANK interaction (depicted in blue arrows) promotes the process through NFATC dephosphorylation, which allows it to enter the nucleus and stimulate gene expression for osteoclastogenesis. RANKL/LGR4 interaction (depicted in orange), on the other hand, inhibits osteoclastogenesis through activation of Gαq-mediated signaling to stabilize GSK3β and prevent its phosphorylation (inactivation). Activated GSK3β phosphorylates NFATC preventing its entry into the nucleus, thus inhibiting osteoclastogenesis. Excessive stimulation of the RANKL/RANK pathway induces *Lgr4* expression as a negative feedback mechanism to finely control the process (11).

In 2016 Luo et al. (11) described LGR4 as a novel receptor for RANKL in the context of osteoclastogenesis. RANK (Receptor-Activator of NFκβ), the known classic receptor for RANKL, induces osteoclast activation upon RANKL interaction. LGR4, on the other hand, inhibits osteoclastogenesis upon RANKL binding, thus tightly regulating bone resorption, which when excessive leads to osteoporosis. In the study by Luo et al., a conditional knockout of *Lgr4* (*Lgr4Fl*/Fl-LysMCre mice) specifically targeting osteoclast precursors, caused increased bone loss and increased activity of osteoclasts as measured by their specific marker TRAP (Tartrate-Resistant Acid Phosphatase). At the molecular level, Luo et al. demonstrated that upon binding RANKL, LGR4 activates Gαq-mediated signaling to stabilize GSK3β and prevent its phosphorylation at Ser9 (i.e. inactivation). Stabilized (unphosphorylated) GSK3β, a known inhibitor of osteoclast differentiation (41), leads to NFATC phosphorylation and its arrest in the cytoplasm, resulting in the deactivation of the osteoclast differentiation program (**Figure 2** **).** By using the ligand binding extracellular domain of LGR4 (LGR4-ECD, aa 28-528), truncated in different regions, Luo et al. deciphered that the fragment containing aa 80-396 specifically interacts with RANKL. They tested the therapeutic potential of LGR4-ECD against osteoporosis, through binding RANKL and thus, preventing its binding to RANK on osteoclasts (see section 6). Very recently, Jang et al. (42) and Ko et al. (43) demonstrated the effects of RANKL point-mutations (RANKL-MT3, having 2 histidine residues in the extracellular domain aa. 220-230 replaced by phenylalanine and tyrosine), which rendered RANKL unable to interact with RANK, but did not interfere with its interaction to LGR4 caused its high affinity to LGR4. As expected, this RANKL mutation had an inhibitory effect on osteoclastogenesis. Both authors showed that when applied *in vitro* or *in vivo* together with the wild-type RANKL, RANKL-MT3 counteracts the effects of the wild-type RANKL and inhibits osteoclast activation. RANKL-MT3 preferably binds LGR4 and reduces NFATC nuclear translocation, a process responsible for osteoclast maturation. Interestingly, Jang et al. (42) demonstrated that RANKL-MT3 administration *in vivo* generates natural anti-wild type RANKL antibodies in mice, therefore acts as a vaccine preventing excessive bone resorption. They also showed that RANKL-MT3 can reverse ovariectomy-induced osteoporosis.

LGR4, however, does not always act as a sponge neutralizing RANKL/RANK interaction. A recent publication by the group of Yue et al. (44) indicated RANKL-RSPO2-LGR4 interaction

in breast cancer cells is crucial for DKK1 production *via* activation of Gαq and β-catenin, enabling further breast cancer metastasis into the bones. Inhibition of this pathway can have a therapeutic potential against tumor spread.

## Extracellular Domain of LGR4 as a Signaling Molecule and a Potential Therapeutic

LGR4 interacts with its ligands- RSPOs, Norrin and RANKL *via* its extracellular domain- LGR4-ECD [also alternatively called LGR4-ED (45)]. LGR4-ECD is a part of the membrane bound form of LGR4, but interestingly, it can also be encountered in nature as a circulating (free) form of LGR4. As such, LGR4-ECD was first described by Hsu et al., who identified a natural Lgr4 splice variant encoding specifically only the ectodomain of LGR4. Hsu et al. (45) showed LGR4-ECD to play an important role as a tight regulator of Wnt signaling during mammalian gonadal development. When administered exogenously *in vitro*, LGR4-ECD inhibited the Wnt signaling in HEK 293 cells in a dose-dependent manner, whereas *in vivo*, LGR4-ECD treatment decreased ovarian development and steroidogenesis in rats. In the paper already discussed above, Luo et al. (11) tested the potential of LGR4-ECD as an anti-osteoporotic drug. They used three mouse models characterized by high levels of bone resorption and osteoporosis, namely ovariectomy, RANKL injection (46), and *Tnfrsf11b-*deficiecy (which leads to a lack of osteoprotective Osteoprotegerin-OPG) (47). In all three models, the authors observed improved bone mass and decreased osteoclast activity in response to LGR4-ECD administration. The fact that LGR4-ECD has an anti-osteoporotic effect indicates its direct interference with RANKL-RANK pathway, which triggers osteoclastogenesis and when excessive, leads to osteoporosis.

**Figure 3:**
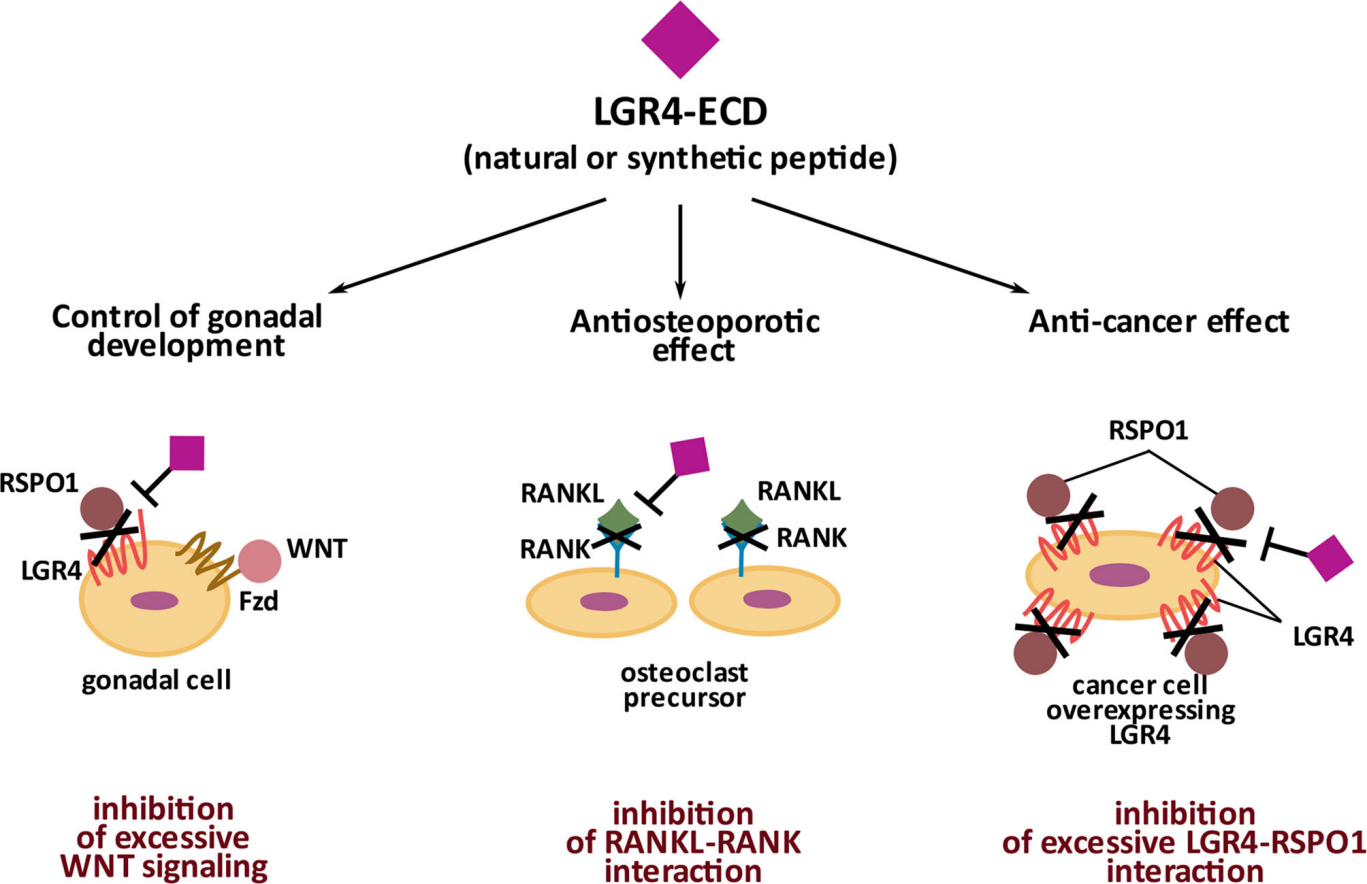
Significance of natural and synthetic extracellular domain of LGR4 (LGR4-ECD). Naturally occurring LGR4-ECD controls gonadal development by binding RSPO1 and preventing its participation in Wnt signaling activation in gonadal cells. Synthetic LGR4-ECD by binding RANKL and preventing its interaction with RANK has an anti-osteoporotic effect. Synthetic LGR4-ECD by neutralizing RSPO1 disables its interaction with LGR4, overexpressed by cancer cells, and thus, holds an anti-cancer potential.

As mentioned before, upregulation of Wnt signaling accompanied by the increased levels of LGR4 and RSPOs marks a variety of tumors. Thus, neutralizing LGR4 activity with LGR4-ECD may have clinical utility in cancer therapy, particularly in the context of new strategies based on checkpoints inhibitors. Such an approach was tested in the study by Tan et al. (48), who demonstrated that RSPO1-LGR4 interaction promotes the immunosuppressive M2 phenotype in tumor-associated macrophages and is associated with a decreased recruitment of CD8+ T cells capable of neutralizing cancer cells. Using either LGR4-ECD or anti-RSPO1 antibody, Tan et al. were able to revert immunosuppression and inhibit the growth of LLC (Lewis lung carcinoma) tumors and B16F10 melanomas. Also, in the paper by Yue et al. (44), LGR4-ECD was shown to hold therapeutic potential against breast cancer metastasis into bones. These studies demonstrate a significant and a tissue context-dependent therapeutic potential of LGR4-ECD, which is summarized in **Figure 3**.

LGR4-ECD mediates its therapeutic effects differentially in different tissues, through binding RSPOs in cancer cells, and binding RANKL in osteoclasts. Therefore, identifying the biding sites for each ligand on LGR4-ECD could enable specificity of its therapeutic effects. In 2013 Wang et al. (12) and then in 2015 Xu et al. (49) described LRRs 3-9 and 4-9, respectively, as the essential domains of LGR4-ECD responsible for binding the furin-like domains of RSPO1). In 2016, by testing 4 differentially truncated forms of LGR4-ECD, Luo et al. (11) concluded that at the primary structure level the region between LRR-NT and LRR8 of LGR4-ECD, corresponding to aa. 28-249, binds RSPO1. Regarding RANKL binding, *in silico* modeling used by Luo et al. predicted that it binds to aa. 108-346 of LGR4-ECD. Data obtained from experiments with 4 differentially truncated forms of LGR4-ECD indicated that RANKL binds to LGR4-ECD in the region within the aa. 80-369 (LRR1-12). Current knowledge on the structural and three-dimensional interaction between LGR4-ECD and NORRIN is very limited. Based on the paper by Deng et al. (25), NORRIN seems to interact with LGR4 *via* its β-sheets 3 and 4 (aa. 95-123) since mutations within these particular fragments of NORRIN impair its interaction with LGR4. However, to the best of our knowledge, there is no information available on specific sites within LGR4 protein structure, responsible for NORRIN binding. Specific LGR4-ECD domains responsible for RSPO1 and RANKL binding are depicted in **Figure 1B** and **Figure 1C**, in the linear and 3D form.

## LGR4: a Negative Regulator of Inflammation

LGR4 has been shown to protect different types of cells against inflammation, most notably in the digestive system. As mentioned above (see section 4), LGR4 protects differentiated hepatocytes against injury (37). By knocking down *Lgr4*, Li et al. demonstrated that LGR4 is essential for hepatocyte survival against TNFα-induced damage in primary cultures and in *in vivo*- models of hepatic injury. The authors demonstrated the activation and internalization of LGR4 upon RSPO1 binding in the hepatocytes. RSPO1 significantly decreased TNFα-induced hepatocyte death, coupled with reduced levels of NFκB-p65 and Caspase-3. Li et al. also showed that RSPO1 protective effects depend on WNT/β-catenin signaling. An earlier study by Planas-Paz (50) described the role of LGR4 and RSPO1 (and ZNRF3/RNF43) in liver zonation and regeneration. The study of Liu et al. indicated *Lgr4* deficiency also leads to more pronounced inflammation (inflammatory bowel disease) of the intestine under Dextran Sodium Sulfate (DSS) administration (51). The authors associated LGR4 deficiency with impaired WNT/β-catenin signaling. Reactivation of WNT/β-catenin signaling with genetic or chemical methods caused a significant reduction of inflammation in the *Lgr4* mutant mice. This study suggests diagnosis of LGR4 levels may have a potential clinical significance in the context of colitis therapy in humans. LGR4 has also been demonstrated to regulate kidney development (52). By knocking down *Lgr4* in mice, the authors revealed that lack of LGR4 during embryogenesis progression promotes increased cell death in the renal peripheral mesenchyme, accompanied by decreased levels of anti-apoptotic protein PAX2 (Paired Box 2). This results in an overall kidney hypoplasia. Wu et al. (53) demonstrated the role of LGR4 in prevention of venous ulcer formation and suppression of inflammation during skin wound healing. In this study, *Lgr4* ko mice exhibited poor skin healing and increased proinflammatory profile within the wounds. They also noted a significant drop in *LGR4* mRNA levels with the appearance of venous ulcers in human subjects, accompanied by an increase in microRNAs 34a and c, directly targeting LGR4 (see section 9) and causing its downregulation at both, mRNA and protein levels. A recent paper by Chen et al. (54) revealed the role of LGR4 in protection against myocardial ischemia-reperfusion (I/R) injury. When *Lgr4* was knocked down in rat cardiac cell line (H9c2), the authors observed increased apoptosis and mitochondrial dysfunction, accompanied by elevated ROS production, decreased ATP production, and inhibition of ERK pathway activation.

A common phenomenon in the studies described above was either impaired Wnt signaling or increased NFκB activity. This seems to be responsible for the detrimental phenotype of *Lgr4* knockout in different tissues. On the other hand, as shown by Ge et al. (55) *Lgr4* overexpression in the osteoarthritic rat synoviocytes, helps diminish the secretion of proinflammatory cytokines [Interleukin 1 (IL1), TNFα, and Interleukin 6 (IL6)] and the activation of NFκB, resulting in an overall decrease in joint inflammation. These studies collectively highlight the clinical potential of *Lgr4* overexpression.

## LGR4 and Regulation of Metabolism in Health and Disease

Besides having anti-inflammatory properties, LGR4 plays an important role in metabolism regulation, acting as a positive or negative regulator of various metabolic cues.

### Circadian Clock

Wang et al. (56) described LGR4 as a linker between circadian clock and production of triglycerides in the liver. By using homozygous *Lgr4* knockout mice (whole body knockout mice, *Lgr4* ^m/m^), these authors showed that LGR4 regulates the circadian oscillations in fat metabolism. The *Lgr4* ko mice had an impaired plasma triglyceride rhythm compared to controls. They also observed circadian clock driven *Lgr4* expression changes in hepatocytes. At the mechanistic level, this study revealed that LGR4 targets Microsomal triglyceride transfer protein (MTTP) to regulate plasma triglyceride oscillations in mice.

### Energy Expenditure

Another study published by the same group (57) focused on the importance of LGR4 in energy expenditure regulation in the adipose tissue and skeletal muscle. By using the same model, as described above-whole body knockout mice, *Lgr4* ^m/m^ (56), as well as combined *Lgr4* and Leptin double knockout (m/m: Ob, a mouse model prone to obesity) mice, Wang et al. demonstrated decreased adiposity and improved glucose metabolism in single *Lgr4* knockout mice, as well as resistance to diet- and double *Lgr4/*leptin manipulation-induced obesity. Mechanistically, *Lgr4* ko mice exhibited a switch from white to brown adipose tissue which caused an increased energy expenditure.

### Glucose vs. Fat metabolism

Another study performed by the same group again on *Lgr4* ^m/m^ mice, described LGR4 as a potential regulator of the glucose to fat metabolism switch in skeletal muscle, according to nutrient availability. Sun et al. (58) showed that when fasted, *Lgr4* ^m/m^ mice exhibit an increase in the expression of lipid oxidation-involved genes and a decrease in GLUT4 (Glucose Transporter Type 4) transporter in the skeletal muscles. These authors (59) also discovered a human gain-of-function *LGR4* A750T (c.2248 G > A) variant, significantly correlating with waist circumference and/or with waist-to-hip ratio in two cohorts- young subjects (≤30y.o) with obesity (BMI >30kg.m^2^) or older subjects (≥40y.o, BMI>30kg.m^2^), both cohorts coming from the population of Eastern China. Zou et al. concluded that the presence of this variant may be contributing to central obesity characterized by abdominal visceral fat accumulation.

### Adipogenesis

Most recently, Dong et al. (60) described RSPO2/LGR4 as critical regulators of progenitor cell differentiation towards adipocytes. In mice, elevated plasma RSPO2 levels were additionally associated with insulin resistance, also plasma RSPO2 levels in humans (specifically in men only) correlated with insulin resistance and fat distribution (elevated RSPO2 levels were associated with obesity). This altogether indicates a negative effect of LGR4 in the adipose tissue.

### Food Intake

LGR4 and its ligands-RSPO1-3 were shown by Li et al. (61) and others to regulate food intake in the brain. The paper by Li et al. and earlier one by Van Schoore et al. (62) demonstrated *Lgr4* is highly expressed in different parts of the rat and mouse brain, respectively. Regions with the highest levels of *Lgr4* are the cortex, hippocampus, amygdala, and hypothalamus, the latter being a central part of the brain regulating appetite and metabolism. By using *in situ* hybridization, Li et al. showed that in the rat hypothalamus, LGR4 is expressed in the VMH (Ventromedial Hypothalamus), regulating hunger (63), namely the feeling of fullness, the ARC (Arcuate nucleus), regulating hunger and satiety (64), median eminence [ME, releasing e.g., GnRH (65)], and the ependymocytes [ependymal cells, supporting neuroglia (66)]. Dark field microphotographs revealed that among these brain structures, ME and ependymocytes have the highest levels of LGR4 expression, followed by VMH and ARC. Data deposited in the Human Protein Atlas (202 human subjects analyzed) also indicate similar trend in humans (with high LGR4 mRNA levels in the hypothalamus). High LGR4 expression in hypothalamic energy homeostatic regions indicates its importance in food intake/metabolism regulation.

Li et al. also identified high expression of RSPOs in the hypothalamic nuclei and suggested RSPOs may have an anorexigenic (food intake-inhibiting) effect in the brain, since their expression is downregulated during fasting and upregulated in satiety states. Similarly, food intake-suppressing effect of LGR4 was observed by Otsuka et al. (67). These authors showed that LGR4 is critical for activation of arcuate proopiomelanocortin (POMC) neurons which regulate food intake.

### Pancreatic Beta Cells

Another metabolic tissue that expresses LGR4 is the insulin-producing pancreatic beta cell (15). However, the functional significance of this receptor in the pancreas remains unknown. Wong et al. (68, 69), and Chahal et al. (70), by manipulating RSPO1- a classical LGR4 ligand, found a controversial role in maintaining beta cell function. In a study published in 2010 by Wong et al. (68) RSPO1 acted as a mitogen for pancreatic beta cells (MIN6 cell line and primary mouse islets) *in vitro*, protecting them against proinflammatory cytokines [IL1β, TNFα, Interferon gamma (IFNγ)]-induced cell death and stimulated insulin secretion, in a glucose-independent manner. As shown by the authors, *Rspo1* expression is regulated by EXENDIN-4 (EX4), an agonist of the glucagon-like peptide-1 receptor, mechanistically stimulating downstream signaling *via* PI3K, in a glucose-, stimulation time-, and concentration-dependent manner. Surprisingly, in the following study by Wong et al. (69) whole body *Rspo1* knockout mice exhibited improved beta cell mass and better glycemic control compared to control mice. Also, when whole body *Rspo1* knockout mice were exposed to STZ in a study by Chahal et al. (70), the *Rspo1* knockout mice were more insulin sensitive, had a lower number of apoptotic β-cells and increased β-cell neogenesis and maturation compared to the wild type littermates. Contradictory findings from the *in vitro* (68) and *in vivo* whole body *Rspo1* knockout mice (69, 70) render RSPO1 controversial in the context of β-cells. None of these reports examine the direct role of LGR4 in β-cells, making it an interesting area for further studies.

### Systemic Metabolic Diseases

In the context of systemic diseases, Li et al. (71) observed a negative correlation between LGR4 levels in the plasma and complications often accompanying type 2 diabetes, such as hypertension. These authors noted plasma LGR4 levels to decline with an increase in blood pressure in the hypertension-suffering subjects with type 2 diabetes, as compared to their non-hypertensive counterparts.

## Mechanisms of Lgr4 Expression Regulation- The Role of miRNAs, RANKL Concentration and Alternative Splicing

So far little is known about mechanisms controlling expression of LGR4. Published data indicate microRNAs, RANKL concentration, and alternative splicing to be important regulators of this process. LGR4 expression at the mRNA and protein levels is controlled by three main microRNAs (miRNAs): 34a and c and miRNA-193-3p. In this context, the study by Cong et al. (72) demonstrated the importance of miRNA34c expression during osteoclast differentiation, showing that miRNA34c suppresses LGR4, (a receptor blocking osteoclastogenesis) and thus enables osteoclastogenesis to proceed. Mechanistically, miRNA34c mediated LGR4 suppression, contributes to increased phosphorylation, and thus, inactivation of GSK3β, which in turn allows NFATC-mediated osteoclast activation. Expression of miRNA34c in osteoclast precursors is regulated by RANKL as well as M-CSF (Macrophage Colony-Stimulating Factor), both stimulating osteoclastogenesis. It is worth emphasizing that negative regulation of LGR4 has an important role in maintaining a basal bone turnover, required for skeleton homeostasis. On the other hand, total inactivation of LGR4 in osteoclast precursors leads to excessive osteoporosis, as described earlier by Luo et al. Besides the bone, miRNA34c, together with miRNA34a orchestrates LGR4 expression in the skin, and in this context, it may have pathological implications. For example, data obtained by Wu et al. (53) indicate miRNA34a and c participate in venous ulcers (pathological skin wound healing) formation *via* negatively targeting LGR4 in keratinocytes. This disables anti-inflammatory potential of LGR4 and thus potentiates skin wound healing pathology. At the molecular level, the miR34-LGR4 axis regulates NFκB signaling pathway. These authors (53) showed that when miRNA-34 a/c levels increase during the pathological skin wound healing, it leads to increased phosphorylation of GSK3β which results in a further phosphorylation of a component of NFκB pathway- p65, at serine 536. This phosphorylation exerts a pro-inflammatory effect. Negative regulation of LGR4 is also mediated by miRNA-193-3p, which by suppressing LGR4 and indirectly LGR4-dependent transcription factor-ATF4 (Activating Transcription Factor 4), tightly controls the process of bone formation (osteoblast differentiation) (73).

Besides microRNAs, *Lgr4* expression can also be modulated by its ligands. For example, Luo et al. (11) demonstrated that expression of Lgr4 in osteoclast precursors is regulated by RANKL concentration and intensity of its interaction with RANK. The authors discovered that when RANKL concentration increases in the extracellular space and it binds RANK to excessively initiate osteoclastogenesis, a negative feedback loop mechanism turns on to stimulate the expression of *Lgr4* **(** **Figure 2** **.)**. This enables a tight control of osteoclast activation. The already discussed paper by Hsu et al. (45), indicated alternative splicing as another *Lgr4* expression regulatory mechanism. They discovered the reading frame shift during *Lgr4* translation, resulting from the introduction of an early termination codon, preceding translation of seven-transmembrane domains of *Lgr4*. Alternative splicing of *Lgr4* leads to formation of a 500 amino acid protein, containing solely its extracellular domain which acts as a decoy receptor for RSPO2 and NORRIN during gonadal development (see section 6).

## Summary

**Figure 4:**
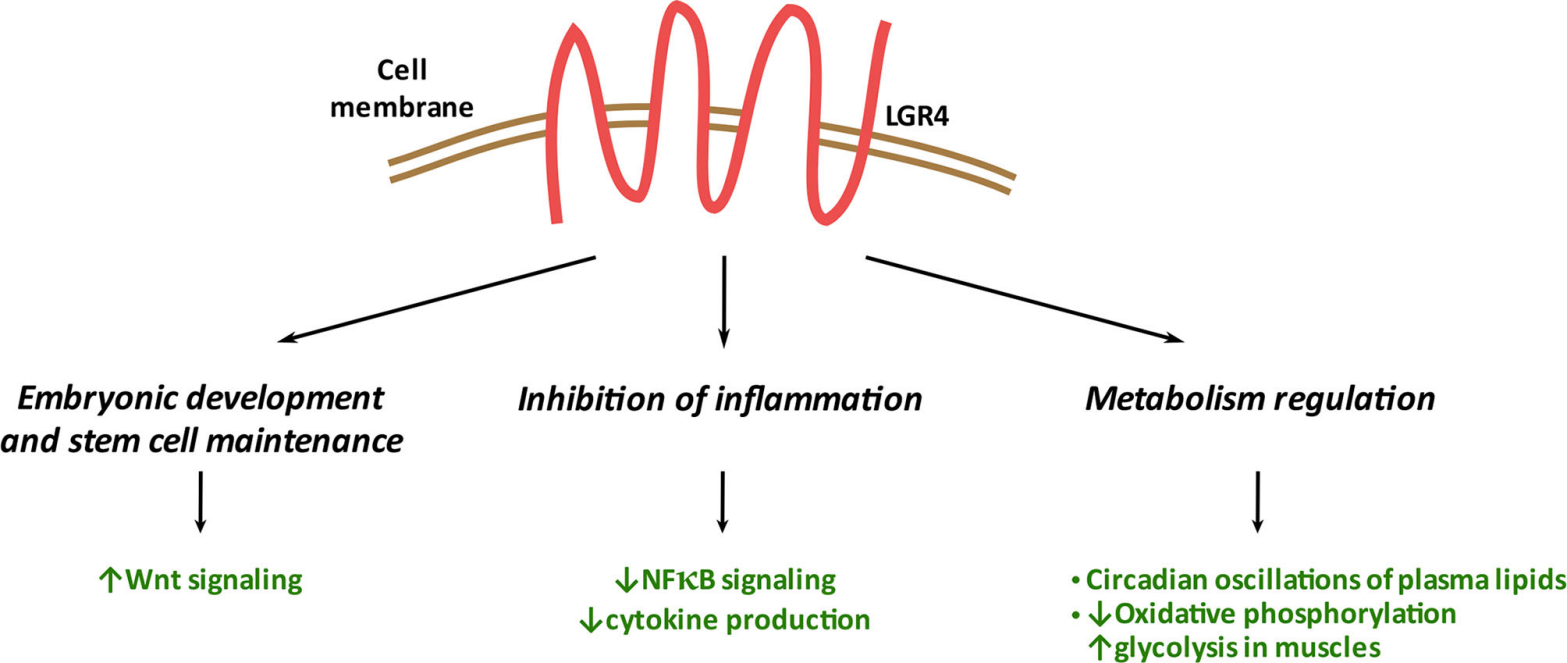
Systemic functions of LGR4. LGR4 plays a significant role in embryonic development/stem cell maintenance through its binding to RSPO1 and regulation of the WNT signaling pathway. LGR4 reduces inflammation in multiple tissues through inhibition of NFκB signaling and cytokine production. LGR4 affects metabolism through multiple mechanisms including circadian clock, oxidative and glycolytic pathways regulation.

LGR4 has emerged as a G protein-coupled receptor that controls multiple pathways and biological processes, traditionally regulating WNT-mediated signaling and crucial during development of multiple organs: eye, reproductive tract, and kidney. LGR4 is required for stem cell maintenance in the small intestine. As recently discovered, it also plays Wnt-independent anti-inflammatory and energy expenditure-orchestrating roles **(** **Figure 4** **)**. Thus, LGR4 is important at a systemic level in the maintenance of whole-body homeostasis. At the molecular level, LGR4 participates in different intracellular signaling pathways, including Wnt, NFκB, TGFβ, and cyclic AMP. This indicates LGR4 to be involved in a plethora of mechanisms controlling cellular events in a tissue-specific manner. LGR4 expression level needs to be tightly controlled: too little or too much of LGR4 can lead to impairments, such as osteoporosis, delayed puberty, obesity, and cancer. Besides the significance of the membrane-bound LGR4, its soluble form- LGR4-ECD exhibits promising therapeutic potential in osteoporosis and cancer/metastasis progression treatment. Altogether, this makes LGR4 an interesting therapeutic target in several clinical contexts. However, the role of LGR4 in many cell types and tissues, such as the pancreatic beta cells, remains to be defined and is an emerging area of interest.

It might be particularly interesting to study this receptor and its regulation in the context of autoimmune and metabolic diseases (e.g., Type 1 and Type 2 diabetes) given the common anti-inflammatory action of LGR4 in different tissues as well as its emerging role in metabolism. Such studies might reveal novel signaling functions and physiological roles of LGR4 and provide new avenues of therapeutic interest. Studies assessing the presence of LGR4-ECD in circulation, and its role in physiological and pathophysiological conditions, might have therapeutic relevance as well.

## Author Contributions

JF prepared the initial and final versions of the manuscript and figures. NGK edited the final version of the manuscript. NLR edited the final version of the manuscript. SD edited the initial and final versions of the manuscript. RCV discussed and edited the initial and final versions of the manuscript and figures. All authors contributed to the article and approved the submitted version.

## Funding

We also gratefully acknowledge our funding sources: Juvenile Diabetes Research Foundation (JDRF)-sponsored postdoctoral fellowship no. 3-PDF-2020-936-A-N (to JF); work in Vasavada laboratory is supported by grants (to RCV) from the National Institutes of Health (R01DK125856, R01DK102893), City of Hope Start-up funds, and the Wanek Family Project to Cure Type 1 Diabetes at City of Hope; work in Dhawan laboratory is supported by grants (to SD) from the National Institutes of Health (R01DK120523), City of Hope Start-up funds, the Wanek Family Project to Cure Type 1 Diabetes at City of Hope, and Human Islet Research Network (NIH) UC4 DK104162.

## Conflict of Interest

The authors declare that the research was conducted in the absence of any commercial or financial relationships that could be construed as a potential conflict of interest.

## Publisher’s Note

All claims expressed in this article are solely those of the authors and do not necessarily represent those of their affiliated organizations, or those of the publisher, the editors and the reviewers. Any product that may be evaluated in this article, or claim that may be made by its manufacturer, is not guaranteed or endorsed by the publisher.
